# What are the key mechanical mechanisms governing integrin-mediated cell migration in three-dimensional fiber networks?

**DOI:** 10.1007/s10237-023-01709-2

**Published:** 2023-06-15

**Authors:** Daniel Paukner, Jonas F. Eichinger, Christian J. Cyron

**Affiliations:** 1 Institute for Continuum and Material Mechanics, Hamburg University of Technology, Eißendorfer Straße 42 (M), 21073 Hamburg, Hamburg Germany; 2 Institute for Computational Mechanics, Technical University of Munich, Boltzmannstraße 15, Garching b., 85748 München, Bavaria Germany; 3 Institute of Material Systems Modeling, Helmholtz-Zentrum Hereon, Max-Planck Straße 1, 21502 Geesthacht, Schleswig-Holstein Germany

**Keywords:** Tissue engineering, Computational modeling, Cell migration, Fibrous networks, Durotaxis

## Abstract

Cell migration plays a vital role in numerous processes such as development, wound healing, or cancer. It is well known that numerous complex mechanisms are involved in cell migration. However, so far it remains poorly understood what are the key mechanisms required to produce the main characteristics of this behavior. The reason is a methodological one. In experimental studies, specific factors and mechanisms can be promoted or inhibited. However, while doing so, there can always be others in the background which play key roles but which have simply remained unattended so far. This makes it very difficult to validate any hypothesis about a minimal set of factors and mechanisms required to produce cell migration. To overcome this natural limitation of experimental studies, we developed a computational model where cells and extracellular matrix fibers are represented by discrete mechanical objects on the micrometer scale. In this model, we had exact control of the mechanisms by which cells and matrix fibers interacted with each other. This enabled us to identify the key mechanisms required to produce physiologically realistic cell migration (including advanced phenomena such as durotaxis and a biphasic relation between migration efficiency and matrix stiffness). We found that two main mechanisms are required to this end: a catch-slip bond of individual integrins and cytoskeletal actin-myosin contraction. Notably, more advanced phenomena such as cell polarization or details of mechanosensing were not necessary to qualitatively reproduce the main characteristics of cell migration observed in experiments.

## Introduction

Cells in living soft tissues are constantly interacting with their surrounding extracellular matrix (ECM) to probe the local mechanical microenvironment. These interactions are known to regulate key processes ranging from biochemical signaling pathways (Nakazawa et al. 2016; Wells 2008) to morphology (Yeung et al. 2005; Ghibaudo et al. 2011) and differentiation (Lee et al. 2013; Yang et al. 2016; Engler et al. 2006). The local mechanical microenvironment also has a tremendous influence on cell migration; it can, for example, determine the mode (Yamada and Sixt 2019) and speed of migration (Wolf et al. 2013), which both play important roles in numerous processes such as development (Franz et al. 2002; Scarpa and Mayor 2016), wound healing (Yue et al. 2010; Gonzalez et al. 2016), and cancer (Friedl and Gilmour 2009; Tozluoğlu et al. 2013; McKenzie et al. 2018).

Cell migration has been studied extensively in two dimensions (reviewed for example in Parsons et al. (2010), Ridley et al. (2003)), since it eliminates a lot of the complexity that comes with three-dimensional fibrous scaffolds such as porosity and microarchitecture (Yamada and Sixt 2019; Cukierman et al. 2001; Charras and Sahai 2014). Systematic studies of moving cells in three-dimensional fibrous networks are challenging, as they require precise control of the network architecture and properties (on the micro- *and* macroscale). Similarly, the majority of computational modeling has focused on 2D so far but more models for the three-dimensional case have been developed in recent years. For example, Moure and Gomez (2018) and Campbell and Bagchi (2021) studied amoeboid migration and Kim et al. (2018) studied adhesion mediated migration in 3D fiber networks. These studies have significantly improved our understanding of the processes underlying cell migration. However, cells in vivo usually migrate in three-dimensional fibrous tissues, and it is unclear whether findings from 2D migration experiments and computational models can simply be translated to 3D.

Another important factor complicating experiments in 3D is the discrepancy between bulk material behavior and local micromechanical properties. Recent studies have pointed out the importance of the local mechanical environment when studying three-dimensional fibrous tissues or scaffolds by showing that the local stiffness a cell perceives can be significantly different from the bulk mechanical properties of the scaffold (Carey et al. 2012; Doyle et al. 2015; Domaschke et al. 2019). For example, it is known that collagen fibers or fibrils have a Young’s modulus on the order of 1 MPa (Jansen et al. 2018), whereas fibrous collagen networks or gels only have a Young’s modulus on the order of 1 Pa−1kPa (Alcaraz et al. 2011; Miroshnikova et al. 2011; Joshi et al. 2018). Different local characteristics such as the mechanical properties of fibers and their arrangement can lead to the same bulk behavior (e.g., Doyle et al. (2015)), which complicates the comparison of experimental results. Influencing factors can be as simple as the source (and therefore exact composition and structure) of the collagen; Wolf et al. (2013) showed that gels made from bovine or rat-tail collagen can lead to similar mechanical behavior during AFM indentation testing (at the same collagen concentration), but to very different pore sizes, which, according to their study, is the rate limiting factor of cell migration in 3D.

In view of the aforementioned difficulties associated with studying cell migration experimentally in three-dimensional fibrous tissues, computational models that capture the mechanical properties of fibrous networks both on the macro- and microscale, can provide additional insights into the fundamentals of cellular migration that are otherwise difficult to assess.

In this paper, we use our previously introduced computational model for three-dimensional cell–matrix interactions (Eichinger et al. 2021) to study cell migration in realistic, three-dimensional fibrous networks. These networks are based on experimentally observed descriptors such as free fiber length, valency, and fiber orientation correlation. They capture the multiscale mechanical behavior of biological hydrogels with fibers having a stiffness on the order of *MPa* and the hydrogel itself a stiffness on the order of 1 Pa−1kPa. The study focuses on integrin-mediated mesenchymal (i.e., contractility-dependent) migration in non-confining networks, i.e., networks with pore sizes larger than the cell nucleus. Because of the large pore sizes and to reduce computational costs, we do not include contact interactions between the cell (nucleus) and the fibers. Additionally, since protease activity only plays a minor role in this case (Wolf et al. 2013), it is not included in the model. To further reduce complexity, biochemical signaling is neglected, and the model is reduced to the three key mechanical players of migration: cellular contractility, focal adhesions, and the transmembrane protein integrin, and the fibrous network. We first validate our model against experimentally observed cell migration speeds and their dependence on fiber stiffness and different pharmacological treatments. By placing the cell in networks with a gradient in stiffness, we identify the key mechanisms sufficient to reproduce durotaxis, the migration of a cell toward higher stiffnesses, in realistic three-dimensional fibrous networks without any prior assumptions on cell polarization making this a so far unique computational model of cell migration in 3D including durotaxis.

## Methods

To simulate cells and their interaction with surrounding matrix fibers we use the computational framework described in detail in Eichinger et al. (2021). In the following, we briefly summarize its main characteristics.

### Network generation

According to Davoodi Kermani et al. (2021), the mechanical properties of collagen gels are mainly governed by their valency, the free fiber length, and the orientation distribution of the fibers. Using stochastic optimization, we are able to match these key metrics to experimental values and therefore are able to create realistic periodic representative volume elements (RVEs) of fibrous collagen networks. For more details on the stochastic optimization algorithm, see appendix A1 in Eichinger et al. (2021). Since the individual fibers of the network experience large deformations during the simulation, we use geometrically exact nonlinear beam finite elements based on the Simo–Reissner theory which are able to capture the most important modes of deformation: axial tension, torsion, bending, and shear. The collagen fibers in our networks are assumed to have a circular cross section with a fiber radius of $90$ nm (Van Der Rijt et al. 2006) and a fiber stiffness $E=1.1$ MPa (Jansen et al. 2018). Individual fibers are connected to form a network by coupling translational as well as rotational degrees of freedom. As was shown in Fig. 5C in Eichinger et al. (2021), this computational approach is able to capture the multiscale mechanics of fibrous networks with individual fibers having stiffnesses on the order of MPa, whereas the resulting macroscopic networks have a stiffness in the range 1 Pa−1kPa. This significant discrepancy between micro- and macroscale is an essential characteristic of fibrous networks and was shown experimentally (Doyle et al. 2015) and numerically for electrospun scaffolds by Domaschke et al. (2019).

In this study, we focus on collagen networks that do not impede migration, i.e., the pore sizes of the network are approximately as large or larger than the cell nucleus. To this end, we generate different kinds of networks which are defined by the total fiber length per volume of the RVE, i.e., line densities with units [μm^-2^]. First, we generate homogeneous networks with a low and high line density and a constant fiber radius of $90$ nm which result in line densities of $3.3 \times 10^{-2}$ μm^-2^ and $4.9 \times 10^{-2}$ μm^-2^, respectively. Second, we use these same networks but with a linearly increasing fiber radius (with a an average radius of $90$ nm) along one coordinate direction to introduce a stiffness gradient. Note that the resulting average collagen concentration can be computed by multiplying the total fiber length per volume with the cross-sectional area of the fibers and dividing by the density of collagen ($v_c = 0.73$ ml/g, Hulmes and Miller (1979)). Doing so, the low and high line density correspond to networks with a collagen concentration of 1.1 mg/ml and 1.7 mg/ml, respectively.

**Fig. 1:**
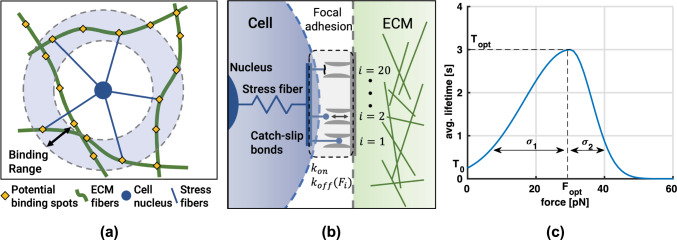
**a** Cell–matrix binding. Once an ECM fiber of the extracellular matrix comes within a predefined binding range, a connection between the cell nucleus and defined binding spots on the ECM fibers can be formed through a stress fiber. **b** Focal adhesion model. A focal adhesion can consist of up to 20 integrins which bind and unbind individually according to a constant on-rate $k_{on}$ ($0.1 \; s^{-1}$, adjusted from Elosegui-Artola et al. (2016)) and a force–dependent off-rate $k_{\rm off}(F)$. **c** Average integrin lifetime. Experimental studies have shown that single integrins show a catch-slip bond behavior (Kong et al. 2009; Weng et al. 2016), i.e., the maximal average lifetime is assumed for tensile binding forces greater than zero

### Cell–matrix interaction

Cells typically attach to the surrounding ECM through transmembrane proteins called integrins. These proteins cluster to form more stable adhesion complexes called focal adhesions which connect the intracellular cytoskeleton to extracellular fibers and allow for a two-way feedback. The cell is able to sense mechanical cues from its environment and at the same time, it is able to apply forces to the attached fibers via contraction of the cytoskeleton. To model these complex interactions, we represent the cell center by a particle which can connect to predefined binding spots (equidistantly spaced with $d_{bs} = 50$ nm (Selhuber-Unkel et al. 2010)) on fibers by creating an elastic connection with a certain probability. This connection can form once a potential binding spot enters a predefined range around the cell (particle) as illustrated in Fig. 1a. The cell radius is assumed to be $R = 10$ μm and the binding range is defined as $R \pm \Delta R$, with $\Delta R = 3$ μm. The resulting connections represent combinations of contractile stress fibers (forming part of the cytoskeleton) and focal adhesions consisting of several integrins (Fig. 1b). Based on experimental results (Kong et al. 2009; Weng et al. 2016), we assume a catch-slip bond behavior for individual integrins, i.e., the bond initially becomes more stable with increased loading and once a certain force is exceeded, bond stability decreases. We approximate the bond lifetime *T*(*F*), depending on the current force *F* in the bond, by the combination of two Gaussian functions (Fig. 1c)

1 $$\begin{aligned} T(F) ={\left\{ \begin{array}{ll} T_\textrm{opt} \exp \left( -\frac{(F - F_\textrm{opt})^2}{\sigma _1^2}\right) &{} \text {if}\ F \le F_\textrm{opt}\\ T_\textrm{opt} \exp \left( -\frac{(F - F_\textrm{opt})^2}{\sigma _2^2}\right) &{} \text {if}\ F > F_\textrm{opt}. \end{array}\right. } \end{aligned}$$

They are defined by the same mean value of $F_{\rm opt}$ but have different spreads $\sigma _1$ and $\sigma _2$, where $\sigma _2$ is prescribed, e.g., by fitting experimental data, and $\sigma _1$ is computed such that the average lifetime at zero force equals some $T_0$. The force–dependent off-rate $k_\textrm{off}(F)$ is then computed as the inverse of the average bond lifetime at force *F*. This results in a simple, generic model for catch-slip bonds with the four parameters $F_\textrm{opt}$, $\sigma _2$, $T_\textrm{opt}$, and $T_0$, which are easy to interpret and identify from experimental data. For the following studies, we used $F_\textrm{opt}=30\; pN$, $\sigma _2=9 \; pN$, and $T_\textrm{opt}=3s$ which is based on data from Kong et al. (2009) and Weng et al. (2016) for the average lifetimes of bonds between fibronectin and $\alpha _5 \beta _1$ integrins. Note that in order to avoid infinite unbinding rates at zero force, $T_0$ must be set to a value larger than 0. Hence, and because of a lack of data from which $T_0$ could be concluded with sufficient certainty, we heuristically choose a value of $T_0=0.25$ s.

Cell contractility is included by allowing existing cell-ECM connections to contract at a constant rate of $\dot{c} = 0.1$ μm/s (Choquet et al. 1997; Moore et al. 2010), naturally limiting the maximum lifetime of a bond which dissolves with increase in probability as the applied load increases. Additionally, we assume that stress fiber cannot contract to a length of less than half the cell radius. Note though that this cutoff is mostly irrelevant since most stress fibers never reach this length since the associated bonds rupture first.

The stress fibers themselves are modeled as elastic springs with a stiffness $k_{SF}$ which we assume to be 1 kPa (Gavara and Chadwick 2016). Once a stress fiber has formed, it contracts with the rate $\dot{c}$, thereby creating a tensile force $F_{SF}$,

2 $$\begin{aligned} F_{SF} = k_{SF} \cdot \Delta x \end{aligned}$$

with $\Delta x$ the length contraction of a stress fiber since its formation. Note that the force points in the direction of the fiber in space. It acts on the cell nucleus (represented by a particle as described above) and the focal adhesion and hence the integrins in that adhesion. The force in an individual integrin, $F_{i}$ is thus

3 $$\begin{aligned} F_{i} = \frac{F_{SF}}{n_{i, \textrm{bound}}} \end{aligned}$$

where $n_{i, \textrm{bound}}$ is the number of currently bound integrins in the focal adhesion associated with the stress fiber. The probability of an integrin unbinding is governed by its load $F_{i}$. The unbinding of the integrins is modeled as a Poisson process yielding an unbinding probability of

4 $$\begin{aligned} p_\textrm{off} = 1 - \exp \left( - \frac{1}{T(F_{i})} \cdot \Delta t \right) \end{aligned}$$

where $T(F_i)$ is computed based on Eq. (1). Note that additional integrins can also bind during that time (further stabilizing the adhesion) with the probability

5 $$\begin{aligned} p_\textrm{on} = 1 - \exp \left( - k_\textrm{on} \cdot \Delta t \right) . \end{aligned}$$

In case all integrins of an adhesion happen to have been dissolved, the entire focal adhesion is dissolved and the stress fiber removed. However, it can form again in the next time steps. The equation of motion of the cell center is the balance between the forces of the stress fibers and a viscous drag force ${{{\underline{\varvec{F}}}}}_\textrm{drag}$ impeding the motion of the cell through the surrounding liquid-filled space:

6 $$\begin{aligned} {{{\underline{\varvec{F}}}}}_\textrm{drag} + \sum _j^N {{{\underline{\varvec{F}}}}}_{SF,j} = {{{\underline{\varvec{0}}}}}. \end{aligned}$$

Here, ${{{\underline{\varvec{F}}}}}_\textrm{drag} = -\gamma \cdot {{{\underline{\varvec{v}}}}}_\textrm{cell}$ with $\gamma$ the friction coefficient of a sphere in a liquid with viscosity $\eta$ based on Stokes Law and ${{{\underline{\varvec{v}}}}}_\textrm{cell}$ the velocity of the cell (center). In our case, we assumed $\gamma = 6 \pi \eta R_\textrm{cell}$ and the viscosity of water $\eta = 1$ mPa$\cdot$s.

The combination of contractility and force–dependent unbinding of individual integrins, which are clustered in focal adhesions, allows our model to realistically capture the distinct lifetimes of integrins (on the order of seconds) and focal adhesions (on the order of minutes). All our simulations were performed using periodic boundary conditions for the RVE (edge length $50$ μm) and the entire computational framework was implemented in BACI (BACI 2021), an in-house finite element code. For more details on the periodic boundary conditions, see appendix A2 in Eichinger et al. (2021). The simulation parameters are summarized in Table 1 in the Appendix.

### Quantitative characteristics of cell motion

Cells are to some extent comparable to random walkers, not moving always monotonously into a specific direction but changing their direction (or even reversing it) over time. Such a random walk-like motion can be characterized in particular by two quantities: on the one hand the velocity of the cells in the three-dimensional space (migration speed), on the other hand by the distance the cells can effectively cover in a given time, which can be characterized by the so-called mean-square displacement (MSD). In case of deterministic motions, both is directly proportional, that is, twice the speed leads to twice the distance covered in a specific period (and thus four times the mean-square displacement). By contrast, for a random motion the relation is more complex. For example, a random walker may move through the space at a very high velocity. However, if that random walker frequently changes or even reverses the direction of motion, it may for a relatively long time remain in the same neighborhood without moving to a truly distant location.

To determine the migration speed, the position $\textbf{x}$ of the particle representing the cell center was extracted from the raw data at (simulated) time intervals of $\Delta t = 60$ s over a total simulated time of $t_{\rm max} = 3600$ s. The displacement between two consecutive data points was computed, which yield the migration speed when dividing by the time interval (i.e., 1 min).

The MSD $\langle r^2(\tau )\rangle$ achieved by cell migration over a time interval $\tau$ was computed as

7 $$\begin{aligned} \langle r^2(\tau )\rangle = \frac{1}{N_{\tau }} \sum _{i=0}^{N_{\tau }} (\textbf{x} (i \Delta t + \tau ) - \textbf{x} (i \Delta t))^2. \end{aligned}$$

Here, $N_{\tau } = (t_{\rm max} - \tau )/\Delta t$ is the maximal number of simulated points in time from which data for the computation of the time average defining the MSD can be harvested for a given $\tau$. To see this, consider, for example $\tau = 600$ s. In that case, the first 50 simulated points in time can be used to compute $\langle r^2(\tau )\rangle$. Because for the associated positions $\textbf{x}(0), \textbf{x}(60\,s), \textbf{x}(120\,s),..., \textbf{x}(3000\,s)$ one has related positions $\textbf{x}(600\,s), \textbf{x}(660\,s), \textbf{x}(720\,s),..., \textbf{x}(3600\,s)$ available in the simulation data to compute (7). By contrast, a position $\textbf{x}(3060s)$ or a position at any later point in time, the associated position required to evaluate the MSD for $\tau = 600\,s$ would be at time $3660\,s$ or later and thus outside the simulated time interval.

### Computational cost

The computational cost varies with collagen concentration, i.e., the number of fibers and thus finite elements used in the simulation. For example, using a collagen concentration of 1.7 mg/ml, there are approximately 2200 elements in the simulation domain. The average runtime of a simulation with baseline parameters is approximately 36 to 48 h on 16 cores (Intel Xeon Platinum 8160). For more details on the computational implementation, see appendix A3 in Eichinger et al. (2021).

### Statistical methods

All simulations were performed with five randomly generated networks (see respective figure captions and legends for specific details). Unless stated otherwise, all data is presented as the average or average (±) standard error of the mean (SEM) of these simulations.

## Results

### Model validation

To validate our model and find its limitations, we first compared it to various experimental results. We focused on the speed of migration, a metric that is most often reported in (three-dimensional) experimental cell migration studies. Our studies were performed in homogeneous networks with a line density of $4.9 \times 10^{-2}$ μm^-2^ corresponding to a collagen concentration of around 1.7 mg/ml.

#### Migration speed

**Fig. 2:**
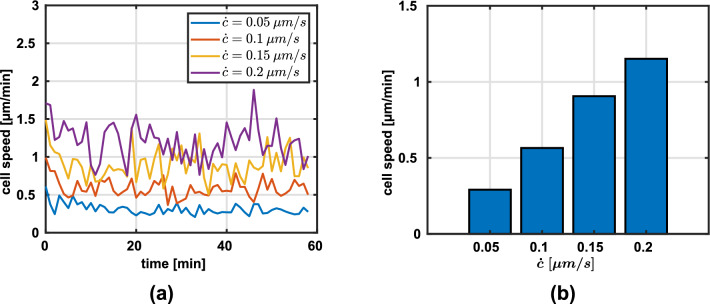
Average migration speeds for varying contraction rates $\dot{c}$ in collagen networks with a line density of $4.9 \times 10^{-2}$ μm^-2^. **a** Simulated migration speed over the range of one hour ($N=5$) **b** Migration speed during the last 30 min of the simulated time ($N=5$, mean)

Reported values of migration speeds in three-dimensional fibrous network experiments are between 0.1 and 10 μm /min (Wolf et al. 2013; Doyle et al. 2015). As can be seen in Fig. 2a, the average migration speed in our simulations (on the order of 1 μm /min) using baseline parameters motivated by experimental data are within the experimentally reported values, especially those of HT1080 and HFF cells. It is important to note that the migration speed is highly cell-type- and substrate-specific. Neutrophils, for example, are reported to migrate with a velocity of $5 - 10$ μm /min (Wolf et al. 2013), whereas HT1080 cells or HFFs migrate at a speed of around $0.5$ μm /min (Doyle et al. 2021). Note that in order to remove any effects of the initialization phase (such as increasing numbers of integrins), we only plot the average speed in the last 30 min in Fig. 2b where migration reached a relatively steady state.

#### Cellular contractility

Through pharmacological treatment, the contractility of cells can be modified. For example, Doyle et al. (2015) and Doyle et al. (2021) used blebbistatin, a myosin II inhibitor, to partially disrupt the contractile apparatus of the cell depending on the dosage. We mimicked the influence of the drug by reducing the contraction rate of the stress fibers and observed a similar qualitative decrease in the resulting migration speeds (Fig. 2b).

#### Varying fiber stiffness

Systematically varying single parameters in a collagen network is challenging in experiments. For example, in Doyle et al. (2015) collagen gels were polymerized at different temperatures. Lower temperatures led to thicker fibers and fiber bundles. However, also the porosity of the gel, which significantly influences migration in 3D (Wolf et al. 2013; Doyle et al. 2015), was found to be temperature-dependent. This example illustrates the difficulties associated with controlling specific mechanical characteristics of biological fiber networks. To overcome this limitation, alternative techniques such as nonelectrospinning (Nain et al. 2009) were developed. Using this technique, Meehan and Nain (2014) and Sheets et al. (2013) placed cells on synthetic fibers and demonstrated that the migration speed is negatively correlated with structural stiffness. It is important to note though that in these experiments, cells migrated along a *single* suspended fiber which limits the general transferability to three-dimensional networks.

**Fig. 3:**
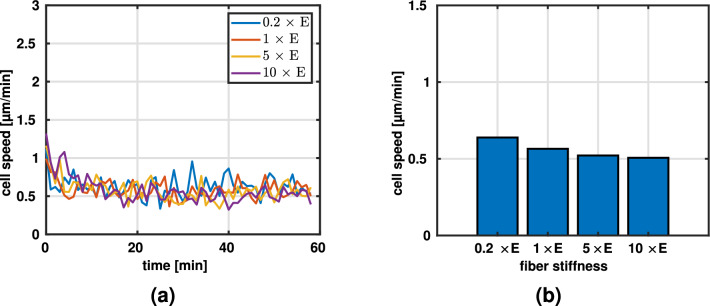
Migration speeds for different fiber stiffnesses (multiples of baseline fiber stiffness $E = 1.1$ MPa). **a** Migration speed during the entire simulation. **b** Average migration speed during the last 30 min of the simulation ($N=5$, mean)

However, since experimental data of the influence of fiber stiffness in three dimensions (without changing other network parameters) is lacking, and because (Doyle et al. 2009) found that cell migration in three dimensions has more in common with migration in one dimension than with migration on two-dimensional substrates, we used the aforementioned studies for a qualitative comparison. They found, for example, that migration on micropatterned 1D substrates and in 3D matrices was dependent on cytoskeletal contraction, whereas 2D migration speed was largely unaffected by a blebbistatin treatment which disrupts the cytoskeleton. The similarity of 1D and 3D migration might arise because migration in 3D is mainly governed by one-dimensional topological cues, the thin fibers making up the network. Based on this, we found a similar trend of decreasing migration speed with increase in stiffness in our simulations, see Fig. 3a and b, respectively. However, we would like to emphasize that this comparison is solely intended to get an idea of how migration in 3D matrices *could* be affected by changes in fiber stiffness because of the lack of experimental data for 3D experiments with varying fiber stiffnesses. As mentioned in section 3.1.1, for Fig. 3b we only plot the average speed in the last 30 min to remove any effects of the initialization phase.

### Integrin turnover

**Fig. 4:**
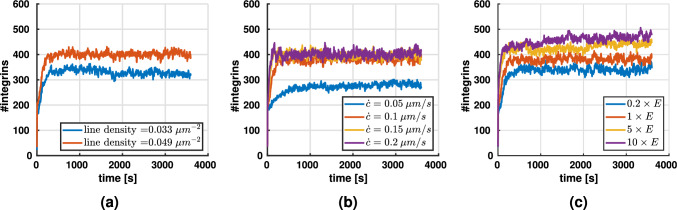
Average number of integrins ($N = 5$ per condition) for varying **a** line densities (contraction rate $\dot{c}= 0.1$ μm/s, fiber stiffness $E = 1.1$ MPa), **b** contraction rates (line density of $4.9 \times 10^{-2}$ μm^-2^, fiber stiffness $E = 1.1$ MPa, and **c** fiber stiffnesses (line density of $4.9 \times 10^{-2}$ μm^-2^, contraction rate $0.1$ μm/s)

Figure 4 presents the number of bound integrins over time. This number stabilizes after an initial rapid increase. The steady state level is influenced by the collagen concentration (Fig. 4a), the contraction rate (Fig. 4b), and the fiber stiffness (Fig. 4c). Interestingly, the number of integrins seems to be insensitive to increases of the contraction rate above 0.1 $\mu m/s$.

### Biphasic mean-squared displacement

Cells placed on two-dimensional substrates of varying stiffnesses have been shown to migrate most effectively on substrates of intermediate stiffness, suggesting a biphasic relationship between the stiffness of the substrate and the mean-squared displacement (Bangasser et al. 2017). The MSD can be interpreted as a measure of how effectively a random walker, in this case the cell, can explore its surroundings.

**Fig. 5:**
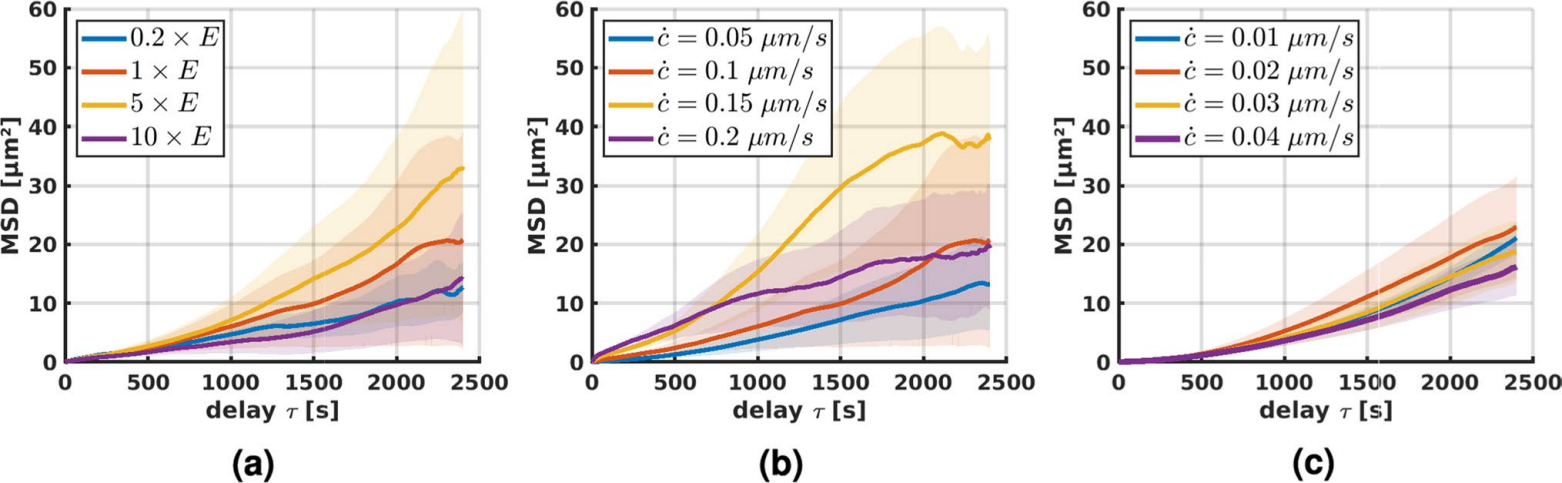
Average mean-squared displacement (± SEM) during the last 40 min ($N = 5$, line density of $4.9 \times 10^{-2}$ μm^-2^): **a** different fiber stiffnesses ($E = 1.1$ MPa) at constant contraction rate $\dot{c} = 0.1$ μm/s; **b** different contraction rates at constant fiber stiffness $E = 1.1$ MPa; **c** different contraction rates at increased constant fiber stiffness $5 \times E$. Note that in this case, the contraction rates were adjusted such that the product of contraction rate and fiber stiffness remains constant compared to **(b)**

To see if we can reproduce this effect in our model, we varied the fiber stiffness at a constant contraction rate. Migration after an initialization phase of 20 min is shown in Fig. 5a. Apparently, also our model produces maximal migration rates at intermediate stiffnesses in agreement with the experimental observations of Bangasser et al. (2017).

In a further set of simulations, we studied the effect of varying contraction rates at constant fiber stiffness, Fig. 5 b and c, respectively. For an intermediate fiber stiffness of $E = 1.1$ MPa (Fig. 5b), we found a biphasic relationship between MSD and contraction rate suggesting that in this regime, cells can increase their migration rate by adjusting their contraction rate (in this case to approximately $0.15$ μm/s). Remarkably, increasing the fiber stiffness by a factor of 5 and adjusting the contraction in such a way that the product of fiber stiffness and contraction rate remained constant compared to the simulations in Fig. 5b, resulted in a biphasic relationship again, albeit less pronounced (5c).

### Durotaxis in three-dimensional fiber networks

**Fig. 6:**
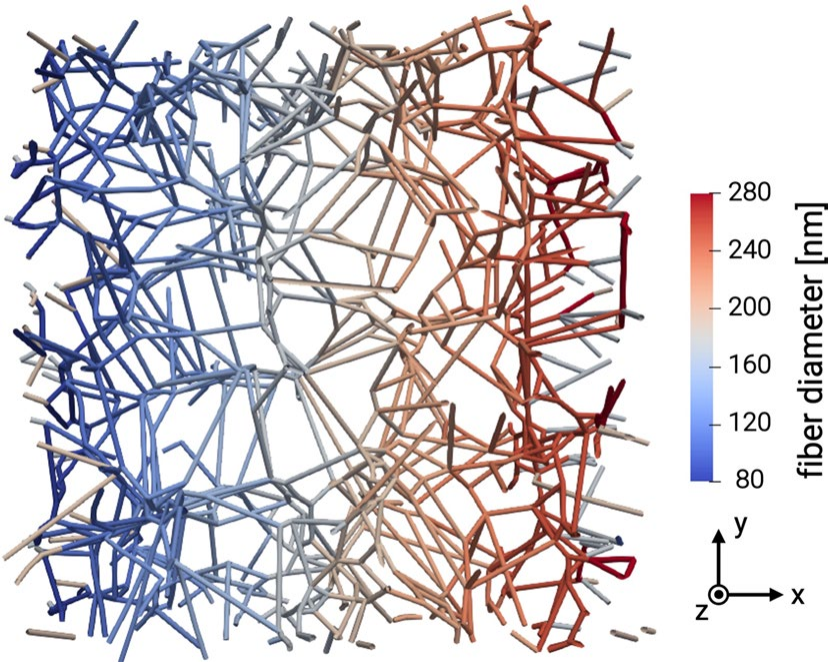
Visualization of a three-dimensional fiber network used to study durotaxis

To study the phenomenon of durotaxis—the preference of cells to migrate toward higher stiffnesses on or in substrates with stiffness gradients—we use exactly the same networks as in the previous sections but introduce a constant gradient of the fiber radius along the *x*-axis of the simulation domain (Fig. 6). All other parameters of the network are kept constant (e.g., line density, network architecture, number of binding spots on the fibers, etc.). Thus, the only consequence of the gradient of fiber thickness is a gradient of stiffness of the fibers in the *x*-direction. We would like to note that the periodic boundary conditions lead to an inconsistency in the x-direction of the domain. However, this approach allowed us to use exactly the same five network structures as in the homogeneous networks with the only difference being the fiber radius distribution and everything else being identical. This way, we can eliminate the possibility that a favorable fiber distribution—potentially occurring at the relatively low collagen concentrations examined—caused preferred migration in the x-direction and ensure that the only factor we changed is the introduction of a stiffness gradient via the fiber radius distribution. To reduce the potential effects of this small boundary region, we adjusted the starting positions of the cells and thereby reduced the chance of the cells reaching those regions during the simulation.

**Fig. 7:**
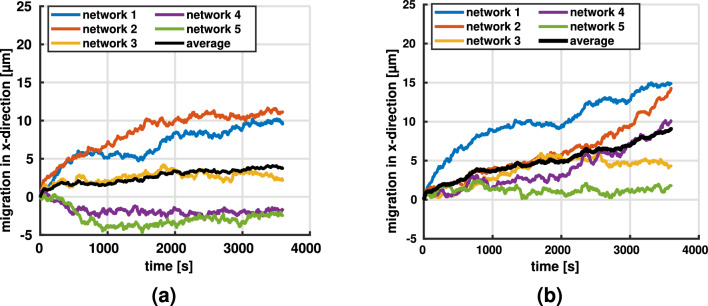
Migration along stiffness gradient (x-direction) for contraction rate $\dot{c} = 0.1$ μm/s and fiber stiffness $E = 1.1$ MPa at a line density of **a** $3.3 \times 10^{-2}$ μm^-2^ and **b** $4.9 \times 10^{-2}$μm^-2^. Mean migration is plotted in black

#### Influence of initial network geometry on durotaxis

**Fig. 8:**
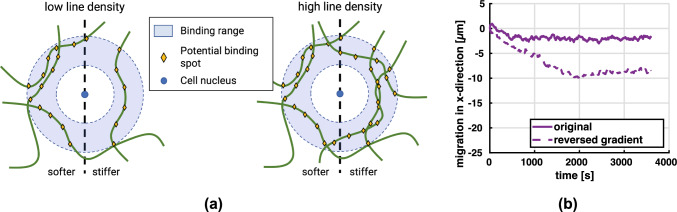
**a** For low collagen concentrations, there is a considerable likelihood that—as a statistical artifact—some cells are located in an environment where they encounter many more binding spots in the direction of decreasing stiffness thus inducing migration against the stiffness gradient. For high collagen densities, the likelihood of such statistical artifacts substantially decreases. **b** The setup of the purple curve in Fig. 7a was reconfigured by inverting the stiffness gradient so that it pointed in negative x-direction. This caused a continued migration in that direction rather than a limited motion in the beginning followed by stagnation

In general, when placed in networks with a stiffness gradient, cells moved preferentially along the stiffness gradient, i.e., in the direction of increasing stiffness (Fig. 7). Interestingly, in a few cases, the migration of cells stagnated from the beginning. In fact, in Fig. 7 one observes for the lower collagen concentration of 1.1 mg/ml (corresponding to a line density of $3.3 \times 10^{-2}$ μm^-2^) that in two cases the cells even first moved slightly opposite to the stiffness gradient before stagnating. We hypothesized that the reason for this behavior is a statistical artifact of the distribution of binding spots around the initial position of the cell. For example, it could happen that the cells encounter in their initial position within their binding range many more binding spots on fibers in the direction of decreasing stiffness (Fig. 8). This would increase the probability of a formation of stress fibers in that direction, eventually leading to some migration against the stiffness gradient that is expected to stagnate, however, quickly because in general the structure of the network would impede such migration. To test this hypothesis, we selected one of the simulations where the cell initially migrated against the stiffness gradient (purple curve in Fig. 7a) and restarted it in a setting, where we had inverted the stiffness gradient by inverting the dependence of fiber radius on the x-coordinate. Indeed, in this reconfigured setup one observes the same cell continuing its motion in negative x-direction (i.e., the direction of the now inverted stiffness gradient) far beyond the point it could reach with a stiffness gradient in positive x-direction. This supports that the systemic driving force is indeed given by the stiffness gradient and that the initial limited motion against it in two cases in Fig. 7a is likely only a statistical artifact resulting from random binding spot availability as illustrated in Fig. 8. Note that this interpretation is supported also by the fact that initial migration against the stiffness gradient appears to happen only in the case of the lower collagen concentration in Fig. 7, where statistical artifacts are more likely. It is worth noting that in several cases in Fig. 7 and also for the reconfigured setup in Fig. 8b one observes after a prolonged period of migration in the direction of the stiffness gradient finally a stage of stagnation. Again this is likely caused by the cell entering a region where—due to a statistical artifact—sufficient binding spots for further migration in direction of the stiffness gradient are missing. Again this interpretation is supported by the fact that such stagnation appears more frequently in case of the lower collagen concentration in Fig. 7 where statistical artifacts are generally more likely than in case of high collagen concentrations. Note that a similar observation of stagnation or slight migration against the gradient has been observed in 2D experiments (Hartman et al. 2016).

**Fig. 9:**
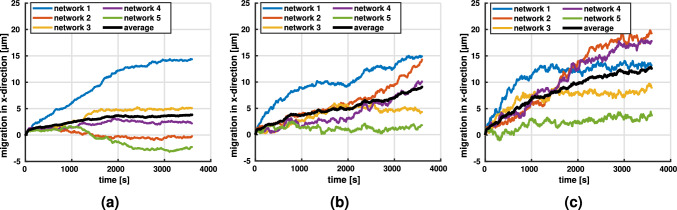
Migration in networks with stiffness gradient in *x*-direction and a line density of $4.9 \times 10^{-2}$ μm^-2^. We studied contraction rates of **a** $\dot{c}= 0.05$μm/s. **b** $\dot{c}= 0.1$ μm/s. **c** $\dot{c}= 0.15$ μm/s with $N=5$ realizations (different colors), respectively. The mean migration rate (black) increases with $\dot{c}$

#### Influence of contraction rate on durotaxis

We studied the influence of the contraction rate $\dot{c}$, which, for example, differs for different cell types, using networks with a line density of $4.9 \times 10^{-2}$ μm^-2^ to reduce random effects of the initial network geometry (see previous section). Apparently, at lower contraction rates, the cells had greater difficulties to sense and follow the direction of the stiffness gradient (Fig. 9), leading more frequently to stagnation or motion opposite to the stiffness gradient. The reason is likely that for lower contraction rates the connection between cells and matrix fibers dissolves with a higher likelihood already before substantial mechanical forces are built up because it takes longer until the regime of optimal survival time of the catch-slip bond is reached. This higher likelihood of dissolution of the cell–matrix connection before it can become mechanically effective can be interpreted as a sort of decreased effective binding spot density, translating into less pronounced directed migration of the cells.

## Discussion

### What mechanisms are sufficient to ensure a balanced adhesion turnover?

Experimental studies have argued that a balanced adhesion turnover is key for efficient cell migration (Huttenlocher and Horwitz 2011; Gupton and Waterman-Storer 2006; Webb et al. 2002). On the one hand, without the formation of stable adhesions, cells cannot attach to the matrix and are therefore unable to move the cell body by cytoskeletal actin-myosin contraction. On the other hand, if adhesions do not disengage, cells might form a large number of long-lasting adhesions resulting in impaired migration (Webb et al. 2004; Chan et al. 2009). Especially for the disengagement of integrins, the contractile apparatus of the cell has been shown to play a major role (Doyle et al. 2015; Vicente-Manzanares et al. 2007). Against this background, it is a key question what mechanisms are required to ensure the balanced adhesion turnover cells need for migration in a manner that is robust across different cellular contractile stresses and different stiffnesses of the extracellular matrix. This question is difficult to answer by experimental studies. Because in such studies it is practically impossible to ensure that not some largely unattended mechanism in the background plays an important role nobody has understood so far. By contrast, in a computational model, one has full control of the mechanisms acting. Therefore, the results shown in section 3 allow us to draw an important conclusion: the interplay of cell contractility (represented in our model by $\dot{c}$) and force–dependent unbinding of adhesions (represented in our model by Eq. (1)) is sufficient to enable the kind of balanced adhesion turnover cells need to migrate. Notably, with these two mechanisms our model robustly reproduced physiological cell migration over a range of collagen densities, fiber stiffnesses, and cell contractilities both in homogeneous collagen networks as well as in networks with a stiffness gradient.

**Fig. 10:**
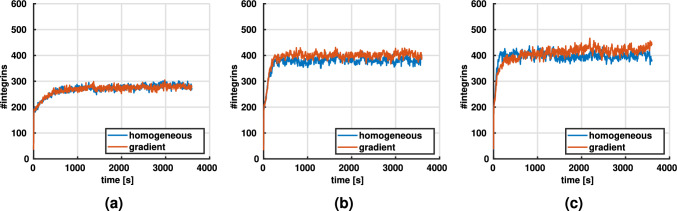
Average number of integrins over time for different contraction rates $\dot{c}$ in homogeneous networks compared to networks with a stiffness gradient ($N =5$, line density $4.9 \times 10^{-2}$ μm^-2^). **a** $\dot{c} = 0.05$ μm/s. **b** $\dot{c} = 0.1$ μm/s. **c** $\dot{c} = 0.15$ μm/s

### How does the cytoskeletal contraction rate affect adhesion formation?

As shown in Fig. 10, the number of integrins through which cells connect to their environment is largely independent on whether they are embedded into a homogeneous extracellular matrix or by contrast one with a stiffness gradient. However, this number of integrins depends on the contraction rate $\dot{c}$ of the cytoskeletal stress fibers. There is some saturation value of $\dot{c}$, above which the number of integrins becomes largely independent on $\dot{c}$. However, below that threshold, the number of integrins decreases as $\dot{c}$ decreases. This can be understood from the characteristic behavior of the catch-slip bond discussed above. When new connections between cells and extracellular fibers are formed through integrins, these connections are initially nearly load-free and thus relatively unstable. The dependence of the bond stability on the transmitted force is often referred to as molecular clutch (Elosegui-Artola et al. 2014, 2018) and illustrated in Fig. 1c. Due to this molecular clutch mechanism, only as the cytoskeleton builds up more and more mechanical loading on the integrin, a more and more stable connection between cell and extracellular matrix forms that is likely to survive for a substantial time. Apparently, for very low cytoskeletal contraction rates $\dot{c}$, there is a considerable period in the beginning when a new integrin connection is established during which that connection is highly unstable and thus may disengage before becoming mechanically effective. The higher the cytoskeletal contraction rate $\dot{c}$, the shorter this critical period becomes. However, above some saturation contraction rate, one expects that nearly all integrins will become mechanically effective before disengaging. Above that saturation rate thus no further effect of increased $\dot{c}$ is expected.

### How does fiber stiffness affect adhesion formation?

A similar argument as presented in the previous section 4.2 may also explain why the number of active integrins increases with fiber stiffness as illustrated in Fig. 4. An increased fiber stiffness may allow the integrins to stabilize more rapidly because for a given cytoskeletal contraction rate it shortens the period during which the integrin-connections are under critically low loading, highly unstable and thus likely to disengage without substantial mechanical effect. That is, increased stiffness of extracellular fibers typically extends the lifetime of integrins and of the respective integrin cluster and focal adhesion, allowing more integrins to bind. We expect that beyond a certain threshold (that generally depends on the cytoskeletal contraction rate), the number of integrins disengaging without substantial mechanical effect becomes negligible so that no benefit can be expected from a further increased fiber stiffness but the tested stiffnesses were not sufficient to show this behavior clearly.

In reality, numerous biochemical signaling pathways are involved in regulating cellular processes such as focal adhesion dynamics or cytoskeletal contractility. Moreover, cells may use several integrin types simultaneously with different binding and unbinding kinetics that will result in different turnover rates allowing a cell to adapt its migration pattern to the given surroundings (Elosegui-Artola et al. 2014). Interestingly, although our model does not capture this complexity in full, it yet reproduces the major experimental findings in cell migration, underlining that at least the fundamentals of this phenomenon can be understood from a surprisingly limited number of factors and processes.

### Biphasic relation between migration efficiency and fiber stiffness

**Fig. 11:**
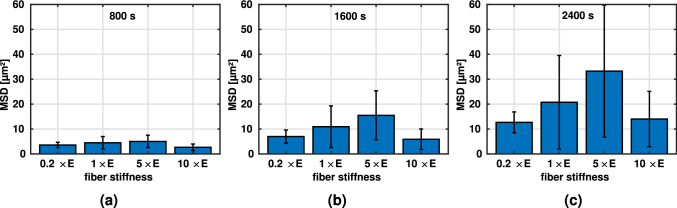
Average mean-squared displacement (MSD) and SEM ($N = 5$) for varying fiber stiffnesses (in multiples of baseline stiffness $E = 1.1$ MPa) at a constant contraction rate of $0.1$ μm/s after **a** 800 s, **b** 1600 s, and **c** 2400 s

Migration speed appears to slightly decrease with stiffness of the extracellular matrix (Fig. 3). However, migration speed itself is not a good measure of how efficiently cells can explore their environment or change their position because it does not account for the fact that stochastic back-and-forth motions may limit this efficiency. Mean-squared displacement (MSD) accounts for this effect and is thus a good measure of the efficiency of cell migration. In line with (Bangasser et al. 2017), we found a biphasic relationship between fiber stiffness and MSD. This suggests that there is an optimal fiber stiffness at which cells can migrate most efficiently, i.e., explore a maximally large portion of their surroundings, given a constant contraction rate and specific integrin dynamics (Fig. 11).

The stiffness maximizing the MSD is likely cell-type dependent as different cell types may exhibit different contraction rates and properties of the integrin catch-slip bonds. It was shown that the optimal stiffness can be modulated by drugs (Bangasser et al. 2017) and potentially also by changing the integrin type and the associated dynamics (Elosegui-Artola et al. 2014).

**Fig. 12:**
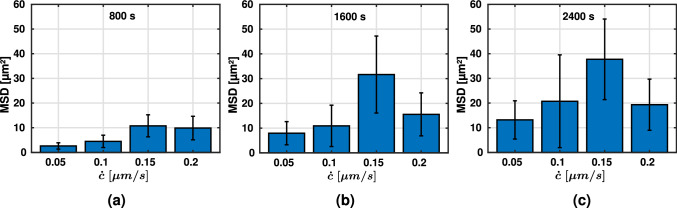
Average mean-squared displacement (MSD) and SEM ($N = 5$) for varying contraction rates and a constant fiber stiffness of $E = 1.1$ MPa after **a** 800 s, **b** 1600 s, and **c** 2400 s

### Biphasic relation between migration efficiency and cellular contraction rate

The MSD depends not only on fiber stiffness but also on the contraction rate via a biphasic relationship as shown in Fig. 5b and Fig. 12 for a fiber stiffness of $E = 1.1$ MPa. This observation was also robust under variations of the fiber stiffness (Fig. 5c). Under such variations, the maximum MSD was achieved if cellular contraction rate and fiber stiffness were chosen such that their product was nearly equal to the one in the baseline case shown in Fig. 5b. This raises the question if there is a more fundamental principle that governs this behavior. For example, there might be a preferred rate of increase of the integrin forces that maximizes the MSD. Possibly, slower rates make focal adhesions too unstable to allow effective cell-fiber interactions, and higher rates make focal adhesions too long-living to allow efficient migration through large spaces. Generally, such complex interactions between adhesion dynamics, contractility, and ECM stiffness are known from experiments (Doyle et al. 2015; Bangasser et al. 2017). Yet, exactly reproducing our computational study in a real experiment is likely challenging because in experiments it is not straightforward to vary single parameters and keep all other parameters constant.

### What causes durotaxis?

One of the first computational studies of durotaxis in three dimensions was proposed by Kim et al. (2018). It relied on a very detailed and complex model where the mechanical interactions of filopodia with a three-dimensional fiber network were used to determine the local effective stiffness the cells perceive (through their filopodia). Using this perceived stiffness, a polarization of the cell was prescribed. Interestingly, our study suggests that details such as the interactions between filopodia and extracellular matrix or polarization are not required to reproduce durotaxis. Rather, the much simpler interplay between contractile stress fibers and a catch-slip integrin bond appear sufficient. To understand, how these two mechanisms alone can produce durotaxis, it is worth revisiting the literature. As was shown by Doyle et al. (2015), Domaschke et al. (2019), Kim et al. (2018), the local micromechanical environment, especially the stiffness a cell effectively perceives, has a significant influence on cellular and subcellular mechanics. Doyle et al. (2015) even showed that the local stiffness perceived by an *individual adhesion* in combination with the contractile forces of the associated stress fiber determines adhesion stability. Durotaxis could thus arise from a spatial difference in adhesion stability: adhesion sites in the direction of the stiffness gradient are loaded slightly more rapidly by cytoskeletal contraction and are therefore slightly more likely to become mechanically effective than adhesion sites in the opposite direction. At the same time, a stress fiber reaching from the cell center toward the stiffer region, i.e., in the direction of the stiffness gradient, can pull the cell slightly more in that direction than a similar stress fiber in the opposite direction because the former interacts with a stiffer matrix. In summary, we observe durotaxis in our model because cell–matrix interactions, based on catch-slip bonds, are more stable in the stiffer regions of the matrix and the contractility leads to larger movements of the cell in the direction of the stiffness gradient. That is, durotaxis can be understood from purely mechanical factors. No prior assumptions on cell polarization or on how stiffness is exactly sensed by the cells are necessary.

### Limitations

Despite the qualitatively very good agreement of our computational framework with various experimental observations, it also has some limitations. As our main objective was to unravel the *mechanical* factors governing cell migration—durotaxis in particular—no biochemical signaling was included. However, it is well known that intracellular signaling pathways are intimately linked to processes such as contraction (Tozluoğlu et al. 2013; Wang et al. 2017; Moujaber and Stochaj 2020) and focal adhesion (dis-)assembly and stability (Sieg et al. 1999; Ren et al. 2000; Huttenlocher and Horwitz 2011) which are the key players according to our findings. These complex interactions were not included in our study. Therefore, when translating the results of our study to in vivo or in vitro studies, one has to keep in mind that there might be important biochemical modulators not yet accounted for in our study. Also, the model for cell–matrix adhesions is simplified and reduced to a single type of integrin. It is known that focal adhesions are made up of numerous proteins. We deliberately neglected that multitude to focus on the essential governing cell migration. However, future studies should include this complexity and examine its role in cell migration. Such studies should also account for the fact that during maturation focal adhesion can grow in size in a way that may depend on external factors such as fiber diameter or fiber alignment (Kim and Wirtz 2013).

Not only the model of cell–matrix interactions in this study was very much simplified compared to reality but also the model of the matrix itself. In our study, we focused on low concentration collagen gels. This allowed us to neglect protease activity which only has a minor influence at low collagen concentrations (Wolf et al. 2013) and would be difficult to include in the discrete beam element model. Additionally, we did not explicitly model the cell nucleus since the pore sizes in our low concentration gels were assumed to be large enough not to impede cell migration. Hence, our computational framework did not include mechanical contact and therefore was not able to capture the effects of confinement which were shown to influence cell signaling and morphology (Balzer et al. 2012) and to modulate migration (Friedl and Gilmour 2009). A further important limitation of the model is the assumed spherical geometry of the cell. In experiments, it was observed that cells have a remarkable ability to change and adjust their shape to their surroundings, see for example (Sheets et al. 2013; Yeung et al. 2005; Thiam et al. 2016). That capability, which is not captured by our model, might allow the protrusions of a cell to explore more of its immediate surrounding and potentially overcome unfavorable binding spot distributions which we observed in low concentration gradient networks, see section 3.4.1.

## Conclusions

In our study, we used a previously developed computational model of cells and extracellular matrix. Cells and matrix were modeled as discrete objects on the micrometer scale. The structure of the matrix realistically mimicked the one of real collagen gels in terms of fiber stiffness and geometric key features (distance between and valency of network nodes, orientation correlation between fibers forming a node). The interactions between cells and matrix were represented by a very simple model relying on two corner stones, the catch-slip bond of the integrins and a contractile rate of the cytoskeletal stress fibers. We demonstrated that these two are sufficient to reproduce the main phenomena of cell migration observed experimentally, including complex phenomena such as durotaxis or the biphasic relation between the mean-squared distance covered by cell migration and the matrix stiffness. Notably, no ad hoc assumptions on cell polarization or details of the mechanical sensing of cells were required. Compared to experimental studies, the great advantage of our computational study is that the set of mechanisms acting can be exactly controlled. This makes the setup of our study particularly suitable to validate hypotheses, which mechanisms are required to produce realistic cell migration. In future work, our model could be extended to include intracellular biochemical signaling such as a dynamic feedback between the integrins and the contraction rate. Additionally, the model could be used to study additional factors known to affect cell migration such as fiber alignment (contact guidance) and the interplay of different types of integrins.

## Appendix A

### A.1 Simulation parameters

**Table 1:** Parameter values

Parameter	Description	Value	References
R	Cell radius	10 μm	Typical value
$\Delta R$	Linking range around cell	3 μm	–
$\dot{c}$	Contraction rate of stress fiber	0.1 μm/s	Choquet et al. (1997), Moore et al. (2010)
$k_\textrm{SF}$	Stiffness of stress fiber	1 kPa	Gavara and Chadwick (2016)
$\eta$	Viscosity (water)	1 mPas	–
E	Young’s Modulus of collagen fibers	1.1 MPa	Jansen et al. (2018)
$R_f$	Radius of collagen fibers	90 nm	Van Der Rijt et al. (2006)
$d_\textrm{bs}$	Binding spot distance on collagen fibers	50 nm	Selhuber-Unkel et al. (2010)
$k_\textrm{on}$	Association rate for integrin	0.1 $\; s^{-1}$	Elosegui-Artola et al. (2016)
$F_\textrm{opt}$	Force at which integrin lifetime is maximal	30 pN	Kong et al. (2009), Weng et al. (2016)
$T_0$	Lifetime of integrin at zero force	0.25 s	–
$T_\textrm{opt}$	Lifetime of integrin at $F_{opt}$	3 s	Kong et al. (2009), Weng et al. (2016)
$\sigma _2$	Spread of Gaussian distribution	pN	Kong et al. (2009), Weng et al. (2016)
$N_{FA,\max }$	Maximal number of focal adhesions per cell	65	Horzum et al. (2014), Kim and Wirtz (2013), Mason et al. (2019)
$N_{i,\max }$	Maximal number of integrins per focal adhesion	20	Changede et al. (2015), Chenget al. (2020)
L	Edge length of RVE	50 μm	–
$\Delta t$	Time step size	0.05 s	–
